# Molecular epidemiological surveillance of viral agents of acute lower respiratory tract infections in children in Accra, Ghana

**DOI:** 10.1186/s12887-022-03419-7

**Published:** 2022-06-24

**Authors:** Anna Aba Kafintu-Kwashie, Nicholas Israel Nii-Trebi, Evangeline Obodai, Margaret Neizer, Theophilus Korku Adiku, John Kofi Odoom

**Affiliations:** 1 Department of Medical Microbiology, University of Ghana Medical school, Korle-Bu, Accra, Ghana; 2 Department of Medical Laboratory Sciences, School of Biomedical and Allied Health Sciences, University of Ghana, Accra, Ghana; 3 Department of Virology, Noguchi Memorial Institute for Medical Research, University of Ghana, Accra, Ghana; 4 Princess Marie Louis Children’s Hospital, Accra, Ghana; 5 Department of Biomedical Sciences, School of Basic and Biomedical Sciences, University of Health and Allied Sciences, Ho, Ghana

**Keywords:** RSV, HMPV, ALRTI, Molecular epidemiology, Children, Ghana

## Abstract

**Background:**

Acute lower respiratory tract infection (ALRTI) in children under 5 years is known to be predominantly caused by respiratory syncytial virus (RSV). In recent times, however, human metapneumovirus (HMPV) has also been implicated. This study sought to investigate and genotype respiratory syncytial virus and human metapneumovirus in children presenting with ALRTIs infection at the Princess Marie Louis Children’s Hospital in Accra, Ghana.

**Methods:**

Children below 5 years who were clinically diagnosed of ALRTI and on admission at the study site were recruited between September 2015 and November 2016 for this study. Demographic data information was obtained by means of a standardized questionnaire; and relevant clinical information was obtained from medical records. Nasopharyngeal swabs were collected from 176 children recruited for the study. Ribonucleic acid was extracted from swabs and cDNA syntheses were performed by RT-PCR. RSV-positive amplicons were sequenced and analyzed for genotype assignment.

**Results:**

RSV and HMPV prevalence among the sampled subjects were 11.4 and 1.7% respectively. Of the RSV positives, 8/20 (40%) were RSV-A and 12/20 (60%) were RSV-B. The highest prevalence was observed in children less than 12 months old. Phylogenetic analysis of the second hypervariable region of the RSV G-gene revealed that all RSV group A viruses belonged to the “novel” ON1 genotype containing the 72-nucleotide duplication; and RSV group B viruses belong to the BA IX genotype.

**Conclusion:**

RSV is frequently detected in children aged under 5 years admitted with ALRTI in Ghana. Continued surveillance of viral aetiological agents is warranted to elucidate the prevalence and transmission patterns of viral pathogens that cause respiratory tract infections among children. This will help inform appropriate intervention approaches.

**Supplementary Information:**

The online version contains supplementary material available at 10.1186/s12887-022-03419-7.

## Background

Acute lower respiratory tract infection (ALRTI) is the leading cause of mortality in children less than 5 years old worldwide. Human respiratory syncytial virus (HRSV) has been known as the main etiological agent of ALRTI [1] in children under 5 years. Advanced molecular techniques have recognized several other newly emerging viruses that also cause lower respiratory infections, one of which is human metapneumovirus (HMPV) [2].

Since it was first detected in 2001 in the Netherlands, infections due to HMPV have been found almost everywhere HMPV has been investigated Studies elsewhere have shown that HMPV does cause both upper and lower respiratory tract infections in young children [3, 4]. Thus, RSV and HMPV have been implicated among viruses as leading causes of childhood lower respiratory tract infection (ALRTI) with severe clinical episodes [5, 6].

There are many viruses known that cause respiratory tract infections in children. However, HMPV and RSV can give very similar clinical presentations. Importantly, both RSV and HMPV belong to the same family Pneumoviridae; RSV being a member of the Orthopnuemovirus genus, and HMPV belonging to the Metapneumovirus genus. They are enveloped and single stranded negative sense RNA viruses. Phylogenetic analysis has revealed two sub-groups for each as A and B [4, 7]. Both viruses infect human populations and produce similar symptoms, which range from mild upper respiratory tract disease like cough rhinorrhea and croup to severe lower respiratory disease such as pneumonia, bronchiolitis, and which may include cough, fever, nasal congestion, erythema and myalgia. Severe cases may have respiratory difficulty, dysphonia, stridor, wheeze and respiratory failure which require hospitalization [5, 8, 9]. Although symptoms from HMPV are generally indistinguishable from those of RSV, they are usually less severe than those of RSV [10, 11]. As such, knowledge of the exact causative agent is always important for targeted therapeutic approaches.

Studies have shown that RSV has seasonal distribution patterns. In temperate countries, the virus circulates in winter and spring seasons, with recurrent outbreaks and epidemics usually occurring in winter [12]. In tropical and subtropical countries also, RSV infection rates are greater in winter, but with considerable regional variations. In Ghana, previous studies showed RSV infection peaks during the rainy seasons of between July to October [13, 14].

The climatic condition in Ghana is tropical, with a dry season in winter and a rainy season in summer due to the African monsoon. The rainy season lasts from May to September in the north, from April to October in the center, and from April to November in the south. However, along the east coast, the rainy season is shorter, spanning April to June, with a break in July and August, and slightly recovers in September and October. The rainiest area is the south. The driest areas are the north, where there’s only one rainy season; and the eastern coast, which includes Accra, where the rainy season is divided into two as indicated above.

Furthermore, in Ghana, some studies have been carried out to determine some viral etiological agents of ALRTIs. Most of the studies done so far mainly focused on the prevalence and characterization of RSV. A few of the studies focused on other viral agents such as parainfluenza and influenza viruses [15]. Studies elsewhere have shown that RSV and HMPV co-infection may increase the severity of ALRTI cases in children [14, 16]. However, the contribution of these two viruses to ALRTI cases seen in Ghana remains to be studied. This study sought to investigate and characterize RSV and HMPV as viral agents of ALRTI among children under 5 years in Accra, Ghana.

## Methods

### Study site

The study participants were recruited from the Princess Marie Louise Children’s Hospital (PMLCH) in the Greater Accra Region of Ghana. The PML is a major children referral hospital within the Ashiedu Keteke sub metro of the Greater Accra region that provides medical care and offers reproductive and child health services to children, individuals and households in Accra and beyond. Accra, in the Greater Accra region where the study was conducted, is the capital of Ghana and lies along the coast to the eastern part of Ghana. As described above, the climatic condition is tropical, has two rainy seasons – a major one in April to June, and a minor season in September and October.

### Participants clinical data and sample collection

Children below 5 years and clinically diagnosed of ALRTI at the study site were recruited for this study between September 2015 and November 2016. With the help of the doctors and nurses, standardized questionnaires and medical reviews were used to obtain participants’ demographic and clinical data, which include age and sex; clinical symptoms such as cough, fever, nasal discharge, fast breathing/ difficulty in breathing, vomiting, diarrhoea, oxygen administration; as well as length of hospitalization. Children with known chronic lung disease, congenital heart abnormalities and asthma were excluded. ALRTI case definition and diagnosis was in accordance with published literature [17–20]. Briefly, ALRTI in a child is defined as a child having a cough or difficulty in breathing, and having one or more of the following: fast breathing, lower chest wall wheezing, in drawing, stridor, or apnoea; with fast breathing defined as > 60 breaths per minute in a child aged < 2 months, > 50 per minute in a child aged 2–11 months, and as > 40 per minute in child aged 12–59 months.

Nasopharyngeal specimens (NS) were then collected from ALRTI diagnosed children as follows: nasal swab was inserted in a depth of about 2 cm into the naris. The swab was rotated gently to collect exfoliates of cells. It was then aseptically removed and inserted into a BD vial containing about 2 ml transport medium (Becton Dickson (BD), USA). The samples were then placed on ice and sent immediately to the Virology unit of the Noguchi Memorial Institute of Medical Research (NMIMR), University of Ghana, where they were kept at -80 °C until they were processed.

### Laboratory analyses

#### Viral RNA extraction and amplification

Ribonucleic acid (RNA) was extracted from about 140 μl of respiratory specimens using the QIAamp viral RNA mini kit (QIAamp viral RNA mini kit, 2014) according to the manufacturer’s instructions. Sterile molecular grade water was used in parallel as negative control in the extraction and all downstream procedures. A two-step RT-PCR method was used for the amplification of partial regions of HMPV F (fusion)-gene and RSV G-gene. Complementary DNA (cDNA) synthesis was performed with 25 μl of RNA extract in a 40 μl mixture containing 200 μM of each deoxynucleoside triphosphate (dNTPs), 5 mM dithiothreitol, 20 U RNasin, a 250 nM of random hexamer primers (Invitrogen- Thermo Fisher Scientific GmbH, Schwerte, Germany), 100 U Moloney murine leukemia virus reverse transcriptase and first-strand buffer containing 250 mM Tris-HCl (pH 8.3), 37.5 mM KCl, and 15 mM MgCl_2_. The reaction was carried out for 5 min at 42 °C, followed by 30 min at 37 °C and finally for 5 min at 94 °C in the Applied Biosytems 2720 thermocycler (Applied Biosystems, Foster City, CA).

A conventional PCR method was employed for the detection and amplification of RSV G-gene and F-gene for HMPV. Amplification of the specified genes were carried out using published primers [21]. The primers used respectively yield 500 bp and 397 bp of RSV A and RSV B fragments out of the 932 bp G gene; and 555 bp and 397 bp of HMPV A and B respectively, out of the approximately 1900 bp or 574 aa fusion protein. Each amplification reaction mixture contained PCR buffer (Invitrogen Germany), 25 mM MgCl_2_, 200 μM dNTPs for RSV / 100 μM dNTPs for HMPV, (Qiagen) 250 nM of each specific primer, 0.5-unit SuperScript^(R)^ III one step RT- PCR System with platinum Taq DNA polymerase (Invitrogen) and 5.0 μl of cDNA in a final volume of 50 μl. The PCR conditions were as follows: for each, initial denaturation was achieved at 94 °C for 5 min, followed by 40 3-step cycles comprising 94 °C for 30 sec, 58 °C for 30 sec and 72 °C for 45 sec for RSV-A; then 94 °C for 30 sec, 53 °C for 45 sec and 72 °C for 60 sec for RSV-B; and 94 °C for 30 sec, 60 °C for 30 sec and 72 °C for 45 sec for HMPV. Semi nested PCR reaction was then carried out using 2 μl of the first round PCR product. All PCRs were performed using the Applied Biosystems Instruments (ABI 2720) thermocycler. The amplified products of 555 bp, 593 bp, 397 bp for the respective viruses were visualized by ethidium bromide staining, following electrophoresis on a 2% agarose gel.

#### PCR purification and cycle sequencing

For sequencing, PCR amplicons were purified using QIAquick PCR purification system (Qiagen, Hilden, Germany) according to the manufacturer’s recommendations (QIAGEN GmbH 2006). In a 96-well plate, 10 μL reactions were then set up, containing 2 μL of the purified amplicon, a sequencing primer at 2 μM, 1x Big Dye buffer (Applied Biosystems), 1 μL of Big Dye Ready Reaction Mix v3.1 (Applied Biosystems), and nuclease-free water. The plate was sealed and pulse centrifuged, after which the PCR was performed under the following conditions: and initial 94 **°C** for 4 sec, followed by 25 3-step cycles at 94 **°C** for 30 sec, 50 **°C** for 15 sec, and 60 **°C** for 4 min. Sequence reaction products were purified with the Agencourt CleanSEQ sequencing reaction clean-up system (Protocol 000600v031, 2006, Agencourt Bioscience, USA). Purified DNA was resuspended in 40ul of nuclease-free water (Life technologies, Ambion, USA). The products were transferred into a 96-well optical plate (Applied Biosystems, USA) and read using a 3130xl Genetic Analyzer (Applied Biosystems, USA) to generate sequence data.

#### Sequence and phylogenetic analyses

Sequence assembly and consensus generation were performed using SeqMan Pro of the Lasergene 8 program suite (DNASTAR, Madison, WI). RSV A and B genomes were assembled using GenBank reference sequences GA1 A2 KT992094 and GB2 CH93-9b AF06521, respectively. Consensus sequences were exported for use in subsequent phylogenetic analyses. Phylogenetic relationships between strains were determined by comparing the sequences with reference sequences, representing various described genotypes, accessed from GenBank (RSVA, *n* = 30 and RSVB, *n* = 44) and aligning them using the alignment programs Align IR V2 and CLUSTAL W. The degree of nucleotide sequence identity and of protein similarity between strains was determined using the default scoring matrices. Phylogenetic relationships between sequences were inferred by the maximum likelihood method with DNADIST/NEIGHBOUR of PHYLIP. The robustness of phylogenies was estimated by bootstrap analyses with 1000 pseudoreplicate data sets generated with the SEQBOOT program of PHYLIP. Phylogenetic trees were constructed using Neighbour-Joining program implemented in PHYLIP. Most of the phylogenetic and molecular evolutionary analyses were performed using MEGA version 7 [22, 23].

### Data analysis

All statistical analyses were performed using IBM Statistical Package for the Social Sciences (SPSS) software 25 (SPSS Inc., Chicago, IL, USA), and GraphPad Prism 7 version 7.04 (GraphPad Software, Inc., La Jolla, California USA). Categorical data were presented as frequencies (percentages). Statistical significance was determined by using the chi-square test with *p* = 0.05. Continuous variables were presented as mean ± SD and significance of difference between groups was tested with independent sample t-test. Binary logistic regression model was used to obtain adjusted odds ratio for risk factors associated with RSV and HMPV positivity.

## Results

### Demographic characteristics of study participants

A total of 188 samples were collected from children under 5 years of age, between September 2015 and November 2016. The number comprised 176 hospitalized patients and 12 non-hospitalized patients – at the emergency ward (Table 1). The results presented here derived from data generated from the 176 hospitalized ALRTI diagnosed children. Our findings showed that respiratory conditions were responsible for approximately 10% of childhood cases necessitating admissions at the Princess Marie Louis (PML) Children’s Hospital, Accra. On average, one out of two (50%) parents or guardians of children with respiratory conditions who were approached consented to be part of the study. As seen in Table 1, the number of hospitalized study subjects comprised 93 males (median age of 10 months and range, 1–60 months) and 83 females (median age of 7 months and range, 1–41 months). In terms of severity of disease, all patients were hospitalized, 12 of which were cases obtained at the emergency departments.

**Table 1:** Demographic characteristics of participants, days spent in hospital and clinical diagnosis with respect to viral types

CHARACTERISTIC /Description	No. of participants n (%)	RSVA Positives n (%)	RSVB positives n (%)	HMPV Positives n (%)	Total Positives n (%)
**Overall Total**	176 (100)	8 (5)	12 (7)	3 (1.7)	22 (13)
**Age groups**					
0–6 months	76 (43)	4 (5)	9 (12)	1 (1)	13 (17)
7–11 months	26 (15)	1 (4)	1 (4)	0 (0)	2 (8)
11–23 months	47 (27)	1 (2)	1 (2)	1 (2)	3 (6)
24–48 months	26 (14)	2 (8)	1 (4)	1 (4)	4 (16)
49–60 months	1 (1)	0 (0)	0 (0)	0 (0)	0 (0)
**Sex**					
Male	93 (53)	2 (2)	6 (7)	0 (0)	8 (9)
Female	83 (47)	6 (7)	6 (7)	3 (4)	14 (17)
**Educational status**					
No school	152 (86)	7 (5)	12 (8)	2 (1)	20 (13)
Crèche	15 (8)	1 (7)	0 (0)	1 (7)	2 (14)
Nursery	8 (5)	0 (0)	0 (0)	0 (0)	0 (0)
Primary	1 (1)	0 (0)	0 (0)	0 (0)	0 (0)
**Patient’s category**					
Hospitalized cases	176 (93)	8 (5)	12 (8)	3 (2)	22 (14)
Not hospitalized (emergency)	12 (7)	0 (0)	0 (0)	0 (0)	0 (0)
**DAYS SPENT IN HOSPITAL** ***N*** **= 176**					
0–3 days	27 (15)	1 (4)	3 (4)	0 (0)	6 (22)
4–6 days	47 (27)	2 (4)	2 (4)	0 (0)	4 (9)
7–9 days	79 (44)	5 (6)	5 (19)	3 (4)	10 (13)
≥ 10 days	23 (13)	0 (0)	2 (9)	0 (0)	2 (9)
**CLINICAL DIAGNOSIS WITH RESPECT TO VIRAL TYPE** ***N*** **= 176**					
Pneumonia	20 (11)	2 (10)	3 (15)	1 (5)	6 (30)
Bronchopneumonia	92 (52)	4 (4)	5 (5)	0 (0)	9 (10)
Bronchiolitis	11 (6)	0 (0)	0 (0)	0 (0)	0 (0)
Unclassified Acute Respiratory Infection (ARI)	53 (30)	2 (4)	4 (8)	2 (4)	8 (15)

### Viral nucleic acid detection

**Fig. 1:**
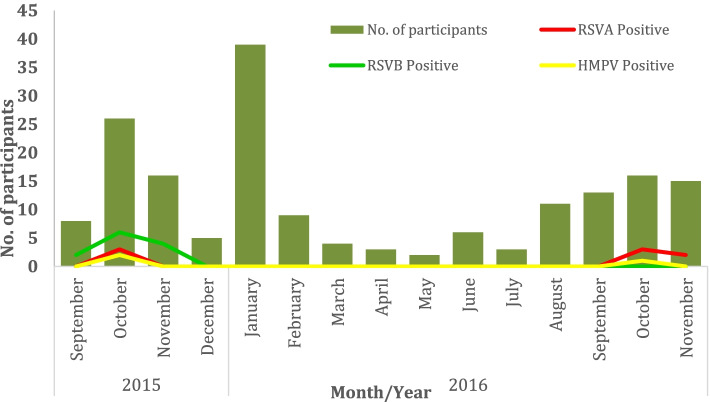
Monthly distribution of RSVA, RSVB and HMPV over the study period. Monthly distribution of viruses detected from samples collected over the period of September 2015 through to November 2016. Vertical bars show the number of participants sampled in the respective months. Coloured lines depict the frequency of detection of the various viruses, with RSV B (green) being highest, followed by RSV A (red) and HMPV (yellow line) in order of magnitude

The number of cases from whom viral nucleic acid was detected were generally low and unevenly distributed over the months within the period of the study (Fig. 1). Even though January was the month with the highest patient number recruited for this study, there were no RSV or HMPV positive patients detected during January to August, but the highest case counts were obtained in October. The frequency of cases was in the decreasing order, RSV B, RSV A and HMPV. Specifically, a total of 23 out of the 176 children (13.1%) studied had either RSV (20/176, 11.4%) or HMPV (3/176: 1.7%) infection. In general, RSV infections were commonly found in children under 6 months, and the number of infections among the children apparently decreased as they approached 5 years of age.

### RSV and HMPV infections among clinically diagnosed cases

Of the children studied, the most common clinical diagnosis made was bronchopneumonia (92/176, 52%), while pneumonia and bronchiolitis were recorded in 20/176 (11%) and 11/176 (6%) children respectively. Fifty-three of the cases studied (53/176, 30%) were those queried, and therefore not classified for specific acute respiratory tract infection cases. RSV A/B- and HMPV- infections were not detected in children with bronchiolitis condition, but infection with these viruses was detected in some of the cases with bronchopneumonia and pneumonia conditions.

### Molecular characterization of viruses

**Fig. 2:**
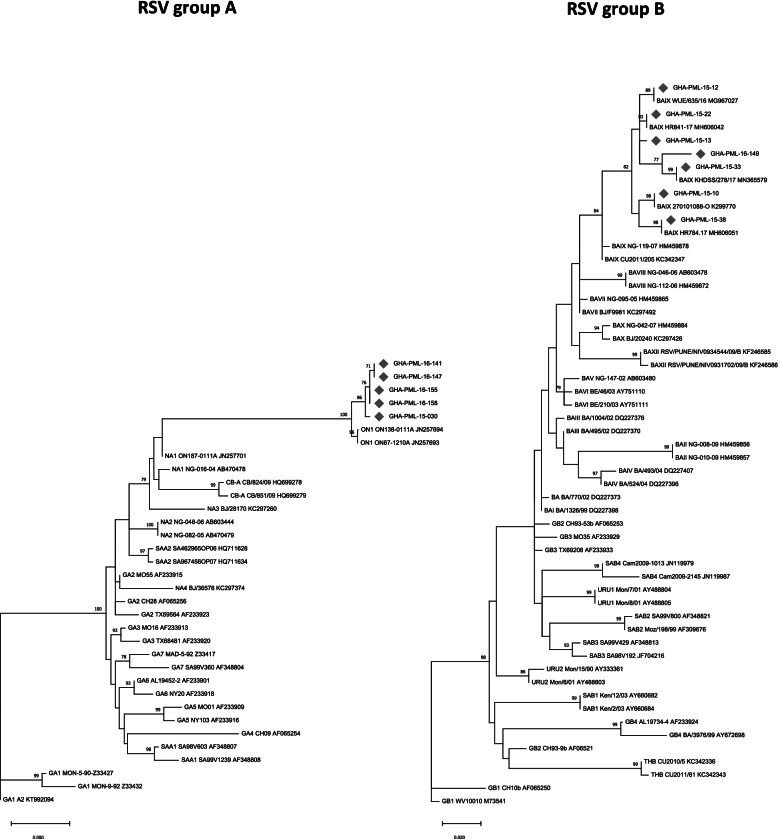
Neighbour-Joining trees representing phylogenetic analysis of RSV genotypes isolated in Ghana between 2015 and 2016. The tree was constructed using the Neighbor-Joining method (Saitou N. and Nei 1987). “The optimal tree with the sum of branch length = 0.90679067 is shown. The percentage of replicate trees in which the associated taxa clustered together in the bootstrap test (1000 replicates) are shown next to the branches (Felsenstein 1985). The tree is drawn to scale, with branch lengths in the same units as those of the evolutionary distances used to infer the phylogenetic tree. The evolutionary distances were computed using the Maximum Composite Likelihood method (Tamura et al.*,* 2004) and are in the units of the number of base substitutions per site. Sequences from this study are shown in bold green color and designated by the geographic location (GHA-PML), patient number and year of collection. The genotype clusters are indicated on the right side of figure. Only bootstrap values greater than 70% are displayed at the branch nodes

The second hypervariable region (VR2) of the G protein gene was successfully sequenced for 5 RSV group A and 7 RSV group B viruses. HMPV sequencing was unsuccessful and therefore its genotype information is not available in this report. The RSV sequences successfully acquired were aligned in MEGA 7 and compared with reference sequences representing the different genotypes. RSV group A strains belonged to the “novel” genotype ON1, whilst RSV group B strains belonged to genotype BA9 (Fig. 2).

### Nucleotide differences analysis

Nucleotide sequence analyses revealed some differences between the RSV-A study samples and ON1 genotype in terms of nucleotide substitutions in the G-gene. Some of the changes were synonymous (namely 66 G → A, and T → C at position 84); others were non-synonymous, which resulted in amino acid alteration (including 95 T → G, 151 G → A, 158 C → T, 260 T → C, 310 T → A). A nucleotide alignment shows these nucleotide changes in more detail (see Additional file 1). With respect to RSV-B, a comparison with the reference BA IX genotype shows at least 20 nucleotide changes between the study and reference sequences. Importantly, all five RSV group B samples had 3 mutations in common at positions 12 (C-T), 292 (C-T) and 301 (C-A). There were also non-synonymous nucleotide changes. Though the direct significance of these mutations could not be established.

## Discussion

Acute lower respiratory tract infections remain a significant public health problem worldwide [8, 19]. Several studies have shown that RSV is a frequent cause of acute respiratory infections in children under 5 years, in whom it contributes up to about 68% of cases investigated. In this study, the prevalence of RSV ALRTIs cases was 11.4%; 75% (15/20) of which cases involved patients less than 1 year old. Elsewhere, in eastern Africa, higher prevalence (22.2%) of RSV has been reported in young Ethiopian infants less than 6 months [24]. Bronchopneumonia, pneumonia and acute respiratory infection were the most reported complications in ALRTI in the present study, which is in agreement with previous study reports [12, 13]. It is however important to note that variations in RSV prevalence may in part be influenced by the detection methods employed. For example, a one-step Triplex Real time RT-PCR employed by You and colleagues to detect HMPV and RSV detection [16]. described RSV and HMPV prevalence of 30.1 and 8.1% respectively; and Adiku and others (2015) also used a conventional multiplex RT-PCR method that obtained a prevalence of 18% for RSV. This study was based on the convectional nested singleplex RT-PCR technique. Factors such as study site, period in the year, duration of study, seasonal variation and sample size used in the study may influence the chances of virus detection.

Of note, the present study analyzed parameters such as age, gender, being in a crowded place such as bedroom sharing with children younger than 5 years, or school/daycare attendance as possible factors that might predispose children below 5 years to ALRTI. No association was found between these variables; most probably due to the relatively small number of patients studied. Nonetheless, infants under 6 months of age were the age group most affected by ALRTIs, which is in line with previous findings [8, 25, 26]. An immature immune system might explain increasing likelihood and severity of RSV with younger age.

The number of admissions between July and October is related to the environmental factors associated with cold temperatures, such as domestic confinement. These conditions are particularly important for the seasonality of viral infections. The study period experienced some rainfalls from the month of October to November 2016 and it was in October that the highest numbers of viral infection cases were recorded, with RSV being more common. It is important to note that other developing countries have also reported a similar phenomenon [8, 9, 15, 20, 27].

Molecular characterization showed that, out of the twenty RSV sequences acquired, all eight (8/20, 45%) RSV A positive strains belonged to genotype ON1; and the twelve RSV B strains (12/20, 68%) belonged to genotype BA9. BA9 genotype, which is the predominant RSV B genotype circulating worldwide, and has also been reported to have circulated in Ghana in 2006 [14]. With respect to ON1, it is worldwide the predominant RSV A genotype and has replaced all other RSV A genotypes [28–30]. This study observed frequent amino acid changes in the ON1 strains of RSV A and BA 9 of RSV B. It is possible that these nucleotide changes over time might constitute accumulation of mutations that eventually confer more advantageous properties to the viruses, and give rise to the emergence of strains with selection advantage [31]. This underscores the significance of genetic variation of RSV and the need for regular monitoring to inform appropriate intervention strategies.

It is important to note that the novel ONI strain is known to be spreading fast across the globe. This strain has been reported in countries such as Kenya [28], Germany [32], Italy [29] and South Africa [33]; and like the BA genotype, it appears to be rapidly replacing all old subtypes [34].

Furthermore, in Ghana, studies thus far have shown that RSV group A and B strains have alternately predominated in two consecutive years or seasons: RSVB was the predominant strain in 2013; RSVA predominated in 2014. Again, in 2015 RSV group B strains predominated, and group A took over predominance in 2016 [12–14]. Hence there appears to be a regular yearly-cyclic-pattern of RSV group dominance. In Belgium, a similar observation was made over 15 consecutive years of RSV surveillance (1996–2011), which revealed a shift from a regular 3-yearly cyclic pattern to a yearly alternating periodicity in which RSV group B is replaced by RSV group A [35]. It is therefore important that further studies involving a larger sample size are required over a couple of years to better understand, or confirm the RSV circulation patterns in Ghana, and for which cause the need for viral genomic characterization cannot be overemphasized.

The study described here has some limitations. First of these is the small sample, and the small number of positive cases confirmed. The duration for the study was also short. These did not allow inferences on seasonal patterns, prevalence, age distributions and genotype distribution to be drawn. The lack of statistical power did not permit any meaningful statistical comparisons regarding dominance among the viruses, and their association with clinical markers. Second is the use of published primers that amplified partial regions of the RSV G gene and HMPV F genes studied. This resulted in shorter sequences that lacked fidelity in phylogenetic analysis. Lastly, we were unable to characterize some of the RSV positive samples as well as the HMPV samples. Future studies would seek to address these limitations.

## Conclusions

This study describes RSV and HMPV as agents of ALRTI in Ghanaian children under the age of five years. RSV was detected more frequently than HMPV. Twenty RSV strains were successfully characterized into group A and B genotypes, indicating a co-circulation of both viral groups. Detailed genomic analysis identified RSV-B as belonging to genotype BA9; whilst RSV A, the most predominant virus genotype found in circulation belonged to the “novel” genotype ON1. These findings further enhance our understanding of RSV and HMPV transmission and epidemiologic patterns in Ghana and thus have the potential to inform measures of controlling these viruses. The study concludes that Respiratory Syncytial Virus and Human Metapneumovirus potentially represent agents of ALRTIs among Ghanaian children below the age of five years. However, the limited size of samples analyzed for this study represents a limitation; and the observed alternating pattern of RSV-A and RSV-B association with ALRTI cases in the studied population would require more years of continued surveillance with a bigger and more appreciable sample size.

## Supplementary Information

**Additional file 1.**

## Data Availability

The nucleotide sequences generated and analyzed in this study have been registered and deposited in the DNA databank of Japan (DDBJ) repository (http://getentry.ddbj.nig.ac.jp/) under the accession numbers LC713043 - LC713047 for the RSV-A isolates; and LC713048 - LC713054 for the RSV-B isolates.
