# Supplementary material for: Molecular epidemiological surveillance of viral agents of acute lower respiratory tract infections in children in Accra, Ghana
Source: BMC Pediatr. 2022 Jun 24;22:364. doi: 10.1186/s12887-022-03419-7 (PMC9229459; doi:10.1186/s12887-022-03419-7)

Additional file 1

(a) Graphic view of nucleotide changes in G genes between RSV-A study samples and RSV-A ON1 reference genotype

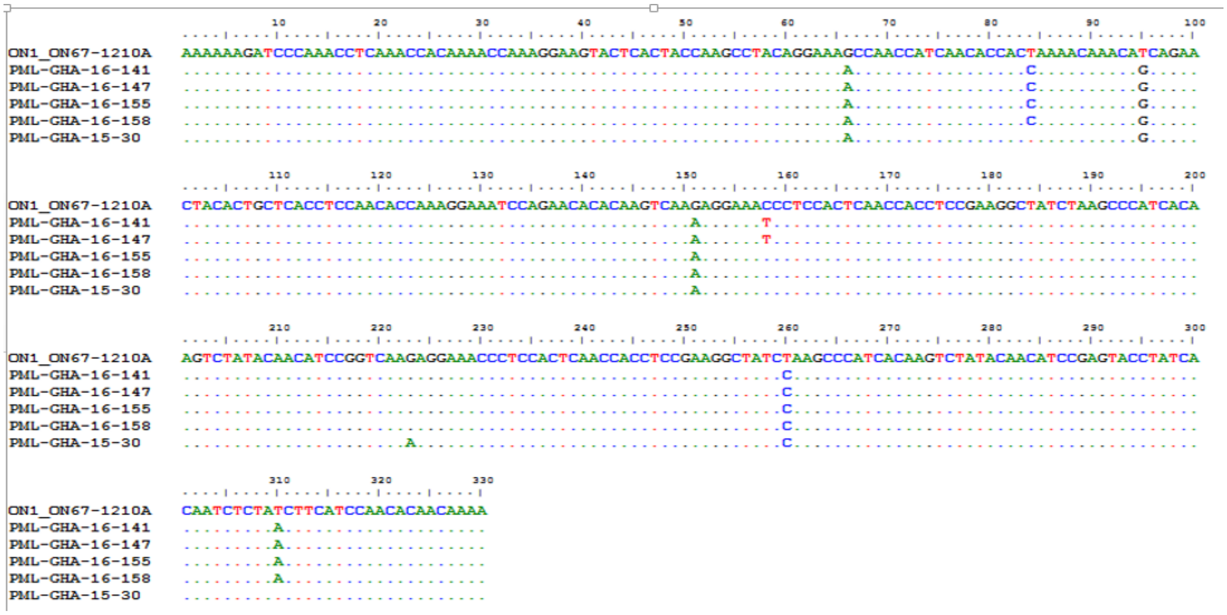

(b) Graphic view of nucleotide changes in G genes between RSV-B study samples and RSV-B BAIX reference genotypes

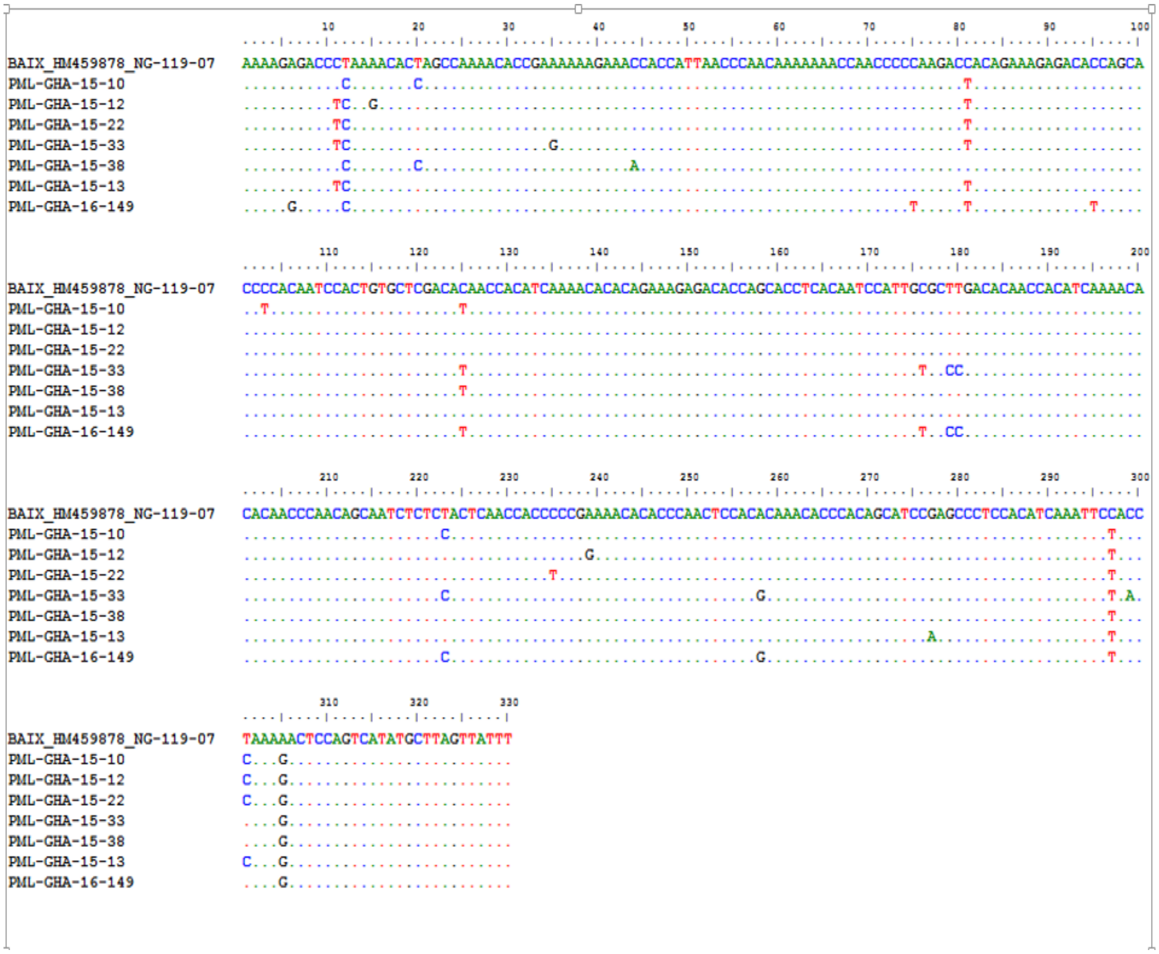

Supplement: Supplementary file 1 — Additional file 1. [file 12887_2022_3419_MOESM1_ESM.pdf]
